# Application of artificial intelligence based on contrast-enhanced CT imaging for predicting peritoneal metastasis in patients with T3/T4 stage gastric cancer

**DOI:** 10.1371/journal.pone.0349614

**Published:** 2026-05-14

**Authors:** Chao Zhang, Siyuan Li, Daolai Huang, Bo Wen, Shizhuang Wei, Yaodong Song, Xianghua Wu

**Affiliations:** 1 Department of Gastrointestinal Gland Surgery, The First Affiliated Hospital of Guangxi Medical University, Nanning, Guangxi, China; 2 Guangxi Key Laboratory of Enhanced Recovery After Surgery for Gastrointestinal Cancer, Nanning, Guangxi, China; 3 Guangxi Medical University, Nanning, Guangxi, China; 4 Department of Obstetrics, Qingdao Municipal Hospital, Qingdao, Shandong, China; Firat University, TÜRKIYE

## Abstract

Gastric cancer, prevalent in East Asia, often presents with peritoneal metastasis at diagnosis, limiting surgical options and reducing survival rates. Given the low sensitivity of current diagnostic methods, this study aimed to develop and evaluate deep learning models based on preoperative contrast-enhanced computed tomography images to improve the detection of occult peritoneal metastasis in T3/T4 stage gastric cancer. We first evaluated the performance of several convolutional neural network architectures and identified Inception-ResNetV2 as the best-performing model. To further optimize the model’s performance, we integrated multiple attention mechanism modules, with the SE module showing the most significant improvement. The SE-augmented Inception-ResNetV2 model achieved a receiver operating characteristic area under the curve of 0.973, Precision-Recall area under the curve of 0.908, and an F1-Score of 0.818, outperforming all other models. Calibration curves demonstrated good agreement between predicted and actual outcomes, while decision curve analysis highlighted the model’s clinical utility. These findings suggest a potential approach for improving clinical predictive modeling by integrating advanced deep learning architectures with attention mechanisms. For patients identified as high-risk, further staging laparoscopy is recommended to minimize unnecessary surgery and guide treatment decisions.

## Introduction

Gastric cancer (GC) is among the most prevalent and lethal malignancies, particularly in East Asia where it often presents at advanced stages [1,2]. Accurate preoperative assessment of disease extent, especially peritoneal metastasis, is crucial for selecting optimal treatment strategies. However, traditional imaging modalities such as contrast-enhanced computed tomography and positron emission tomography-computed tomography frequently struggle to detect small or subtle peritoneal lesions, resulting in a significant proportion of patients being diagnosed with PM only during surgery [3–5]. Although staging laparoscopy can reliably identify occult PM, its clinical application is limited by high costs, restricted accessibility, and its invasive nature [6,7]. Consequently, developing more reliable, non-invasive approaches to diagnose and predict peritoneal metastasis remains a critical clinical priority, particularly for patients with advanced T3/T4 stage gastric cancer.

In recent years, artificial intelligence (AI) techniques—especially deep learning methods—have demonstrated significant potential in various domains of oncologic imaging [8]. AI and radiomics approaches can extract quantitative and high-dimensional features from CT images—capturing subtle textural and morphological patterns that are imperceptible to human vision [9–11]. Convolutional neural networks (CNNs) particularly excel in automated feature extraction and classification tasks, minimizing reliance on manual feature engineering [12]. Several studies have illustrated the adaptability of CNN-based models in identifying gastric neoplasms, stratifying lymph node involvement, and predicting patient prognosis using histopathological images [13,14]. For instance, Wang et al. applied deep learning on resected lymph node histopathology images to predict postoperative outcomes, highlighting the capability of CNNs to capture nuanced features overlooked by conventional methods [15]. Similarly, Cho et al. demonstrated that automated CNN models effectively classified gastric neoplasms in endoscopic images with high specificity, indicating their potential for generalization across diverse imaging modalities [16]. More specifically, deep learning approaches for PM prediction have also begun to emerge. Jiang et al. developed a model employing densely connected CNN architectures to detect occult PM in gastric cancer patients, achieving promising area under the receiver operating characteristic curve (ROC-AUC) values across multiple validation sets [17]. Mirniaharikandehei et al. integrated machine learning classifiers with handcrafted features from CT images, obtaining relatively high specificity but moderate sensitivity in identifying peritoneal metastasis [18]. Despite these advancements, many studies encompassed patients across a broad spectrum of T stages or lacked fine-grained attention strategies, potentially limiting the precise detection of subtle peritoneal spread.

To address these limitations, researchers have increasingly adopted attention mechanisms to enhance CNNs’ ability to focus on the most salient regions or channels within an image [19,20]. Modules such as the Squeeze-and-Excitation (SE) block, the Convolutional Block Attention Module (CBAM), and other attention mechanisms have exhibited superior performance across multiple medical applications, including medical image classification, lesion detection, and disease diagnosis [21–23]. By recalibrating feature maps, attention mechanisms can emphasize critical tumor regions or peritoneal surfaces that might otherwise be overlooked by conventional convolution layers [24,25]. Although attention-based approaches have demonstrated potential in gastrointestinal endoscopy tasks—such as polyp detection or lesion segmentation—their systematic application in CT-based PM prediction for advanced gastric cancer remains underexplored [26]. Furthermore, the clinical utility of these methods extends beyond raw classification metrics such as ROC-AUC, encompassing factors like precision–recall trade-offs, calibration reliability, and decision curve analysis (DCA) to assess net clinical benefits [26,27]. Integrating attention mechanisms into CNN frameworks and assessing their performance using comprehensive evaluation metrics may thus yield a more robust and clinically relevant approach for detecting peritoneal spread [28,29].

Building on these insights, our study specifically targets T3/T4-stage GC patients, who face the highest risk of peritoneal metastasis, and employs an Inception-ResNetV2 architecture augmented with an SE attention mechanism. This architecture is designed to capture both fine-grained local details and broader global context from CT images, thereby potentially enhancing sensitivity to subtle peritoneal deposits. We additionally mitigate class imbalance using focal loss and conduct a comprehensive model evaluation across multiple performance metrics, including ROC-AUC, precision–recall AUC (PR-AUC), F1-score, calibration curves, and DCA. This study aims to construct a high-performance predictive model for peritoneal metastasis in advanced gastric cancer through a systematic evaluation of various CNN architectures and attention mechanisms. By determining the optimal approach, we aim to improve risk stratification and facilitate personalized clinical decision-making. Moreover, by validating the clinical utility of threshold optimization, our model serves as a practical, non-invasive tool to minimize unnecessary laparotomies and optimize patient management in clinical practice.

## Materials and methods

### Study design and patients‌‌

**Fig 1:**
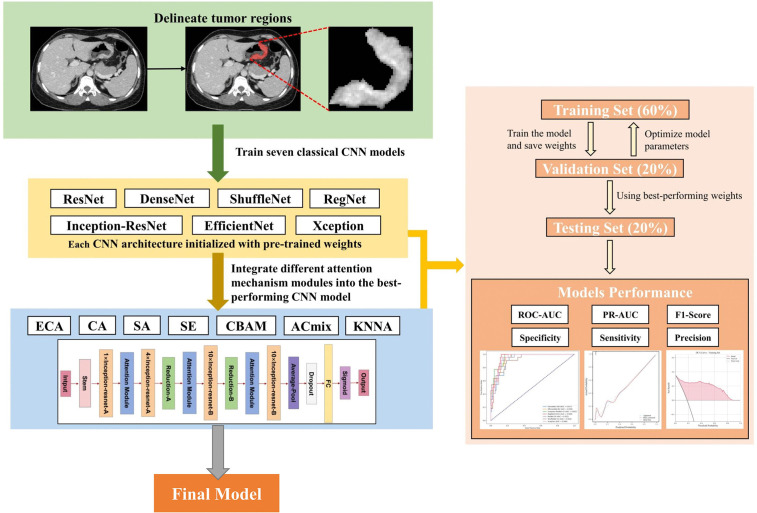
Flowchart of the research process. The study begins with the delineation of tumor regions from contrast-enhanced CT images. These images are used to train seven CNN architectures: ResNet, DenseNet, ShuffleNet, RegNet, Inception-ResNet, EfficientNet, and Xception, with each architecture initialized using pre-trained weights. The best-performing CNN model, Inception-ResNetV2, is then further enhanced by integrating various attention mechanism modules, including ECA (Efficient Channel Attention), CA (Coordinate Attention), SA (Spatial Attention), SE (Squeeze-and-Excitation), CBAM (Convolutional Block Attention Module), ACmix (Attention Convolution Mix), and KNNA (K-Nearest Neighbors Attention). The dataset is divided into training (60%), validation (20%), and testing (20%) sets. Model performance is evaluated using ROC-AUC, PR-AUC, F1-Score, sensitivity, specificity, and precision metrics. The final optimized model demonstrates superior performance in predicting peritoneal metastasis in patients with advanced gastric cancer.

In this retrospective study, we included 585 patients diagnosed with T3/T4 stage gastric cancer at the First Affiliated Hospital of Guangxi Medical University from March 2017 to March 2024. The cohort comprised 478 patients without peritoneal metastasis and 107 with the condition. The data for this study were accessed on August 5, 2024. We collected venous phase contrast-enhanced CT images and clinical data for all participants, with detailed demographic and clinical characteristics presented in Table 1. The inclusion criteria for the study were as follows: (1) newly diagnosed patients who had not undergone chemotherapy or other neoadjuvant treatments prior to surgery and had no history of other malignancies; (2) preoperative evaluation performed according to the 8th edition of the American Joint Committee on Cancer guidelines, indicating the feasibility of curative surgery; (3) high-quality abdominal CT images with clear visualization and no significant noise interference, along with complete clinical data; (4) clear clinical staging provided by both contrast-enhanced CT and endoscopic ultrasound, with no obvious signs of peritoneal metastasis or ascites detected by radiologists in the CT images; and (5) thorough examination of the abdominal cavity during surgery by the surgeon, with pathological confirmation of any suspicious peritoneal lesions to determine the presence of peritoneal metastasis. Patients were randomly divided into training, validation, and test sets in a 6:2:2 ratio to develop and validate the predictive models. The study adhered to the Declaration of Helsinki and received approval from the Ethics Committee of the First Affiliated Hospital of Guangxi Medical University, with the approval number (2024-E550-01). Due to the retrospective nature of the research, the requirement for informed consent was waived by the ethics committee. A detailed overview of the research process is presented in Fig 1.

**Table 1:** Characteristics of patients with gastric cancer in training, validation and test set.

Characteristic		Training set (349)	Validation set (118)	Test set (118)	*P* value
Age, mean(SD)		56.15(12.20)	56.4(11.53)	55.69(11.51)	0.895
Gender	Male	233 (66.76%)	81 (68.64%)	74 (62.71%)	0.605
	Female	116 (33.24%)	37 (31.36%)	44 (37.29%)	
Peritoneal Metastasis	Absent	286 (81.95%)	96 (81.36%)	96 (81.36%)	0.984
	Present	63 (18.05%)	22 (18.64%)	22 (18.64%)	
Major location	Cardia	154 (44.13%)	49 (41.53%)	47 (39.83%)	0.861
	Body	157 (44.99%)	59 (50.00%)	57 (48.31%)	
	Antrum	28 (8.02%)	6 (5.08%)	9 (7.63%)	
	Whole	10 (2.87%)	4 (3.39%)	5 (4.24%)	
Biopsy differentiation	Well or moderate	66 (18.91%)	16 (13.56%)	19 (16.10%)	0.385
	poor	283 (81.09%)	102 (86.44%)	99 (83.90%)	
Clinical N stage	N0	81 (23.21%)	16 (13.56%)	15 (12.71%)	0.091
	N+	268 (76.79%)	102 (86.44%)	103 (87.29%)	

### CT examination and image segmentation

All patients underwent abdominal contrast-enhanced CT examinations within two weeks prior to surgery, with equipment and acquisition parameters detailed in S1 Table. Patients fasted overnight and consumed 500–1000 ml of water before scanning to ensure the stomach was sufficiently distended for clear gastric wall visualization. Scans were conducted in the supine position with breath-holding to reduce respiratory motion artifacts. Venous phase images were captured using the Picture Archiving and Communication System. Tumor segmentation was performed with ITK-SNAP software (version 4.0.2, http://www.itksnap.org/). For each patient, the slice with the largest tumor cross-section was identified, and a region of interest (ROI) was drawn on this slice. If the largest cross-section was difficult to determine, ROIs were drawn on three consecutive slices, and the slice with the largest cumulative pixel area across the masks was selected as the slice with the largest ROI. The segmentation process was assisted by two radiologists, each with five years of experience in abdominal CT interpretation. The radiologists involved in ROI delineation were only aware of the gastric cancer diagnosis and were blinded to the patients’ imaging and histopathological staging to minimize potential bias.

### Image pre-processing

We performed image preprocessing to address variations in scanning protocols and patient factors that might lead to inconsistent intensity distributions. We first adjusted the window width to 350 and the window level to 40 for each CT image, which is a commonly used setting in abdominal CT to achieve adequate contrast for evaluating soft tissues and potential tumor boundaries. Subsequently, a Z-score normalization was applied across the entire dataset to mitigate inter-scan variability in intensity values. From each preprocessed slice, we then cropped a square bounding box encompassing the ROI and resampled the images using the Lanczos interpolation method to match the specific input resolution required by each model (e.g., 299 × 299 pixels for Xception and Inception-ResNetV2, and 224 × 224 pixels for other architectures). Each processed image underwent thorough visual inspection to ensure accurate preservation of the ROI and to exclude any significant artifacts.

### Development of CNN models

CNNs have been widely adopted in medical image analysis due to their powerful feature extraction and pattern recognition capabilities. Compared to Vision Transformers, CNNs have fewer parameters and utilize convolution and pooling operations to capture local features and achieve spatial invariance [30], making them more suitable for processing small-scale datasets such as medical CT images [31]. Therefore, this study evaluated various CNN architectures, including ResNet50, DenseNet169, EfficientNet B2, Xception, RegNet(X-032), ShuffleNet, and Inception-ResNetV2. Each model offers unique advantages: ResNet alleviates the vanishing gradient problem in deep models by introducing residual blocks, thereby improving training efficiency and accuracy [32]; DenseNet enhances feature reuse and gradient flow through densely connected layers, making the model more efficient [33]; EfficientNet achieves an optimal balance between network depth, width, and resolution through compound scaling, resulting in an excellent performance-to-efficiency ratio [34]; Xception employs depthwise separable convolutions, significantly improving parameter efficiency and computational performance [35]; RegNet introduces a configurable architecture generator, allowing the design of networks that adapt to various task requirements [36]; ShuffleNet optimizes the computational efficiency of lightweight networks through channel shuffling, making it highly suitable for applications with limited resources [37]; ShuffleNet optimizes computational efficiency for lightweight networks through channel shuffling, making it highly suitable for mobile applications [38]. By incorporating a diverse range of classical CNN models, we aimed to comprehensively evaluate and optimize the performance of deep learning models in predicting peritoneal metastasis.

### Training and optimization of CNN models

During model training, we initially trained the models using the training dataset, adjusted parameters based on the validation set performance, and conducted final evaluations using the test dataset. We employed transfer learning by initializing with pre-trained ImageNet weights to enhance learning efficiency and performance [39].

Due to the complexity of deep learning models and the tendency for overfitting in small datasets, we employed various data augmentation techniques on the grayscale CT images, including random horizontal flipping, vertical flipping and rotation. Each augmentation technique was applied to each image sample with a 50% probability to simulate different tumor positions and gastric morphologies, effectively increasing the diversity of the training data and enhancing the model’s generalization ability [40]. Further, regularization optimizers, dropout layers, and early stopping strategies were employed to prevent overfitting. Regularization optimizers and dropout layers help prevent the model from overfitting the training data [41,42], while early stopping monitors the model’s performance on the validation set and stops training when overfitting begins [43]. The initial learning rate was set at 0.001, using the AdamW optimizer with a batch size of 4, and dropout probabilities of 0 and 0.3. The loss function employed was BCEWithLogitsLoss.

### Integration of attention mechanisms to enhance model performance

Traditional CNN models may overlook key local features when handling class-imbalanced or fine-grained classification tasks. To address this issue, various attention mechanisms were integrated into the Inception-ResNetV2 model to further improve the classification performance. Attention mechanisms guide the model to focus on important regions of the image while ignoring irrelevant or noisy information, thereby enhancing classification accuracy and robustness [44]. After determining that the Inception-ResNetV2 model exhibited the best overall performance among the base models, we chose to integrate different attention mechanism modules into this model to further optimize its performance. We experimented with several attention mechanism modules, including Attention Convolutional Mix (ACmix), CBAM, Coordinate Attention(CA),Efficient Channel Attention (ECA), K-Nearest Neighbors Attention (KNNA), SE and Shuffle Attention(SA). ACmix enhances feature representation by integrating both local and global information while maintaining computational efficiency [45]. CBAM combines channel and spatial attention mechanisms to adaptively reinforce important feature regions [46]. CA captures long-range dependencies while preserving spatial positional information by incorporating coordinate information [47]. ECA improves feature selection capability through an efficient channel attention mechanism, avoiding increased computational complexity [48]. KNNA identifies and strengthens the relationships between similar features using a k-nearest neighbors mechanism [49]. The SE module enhances the model’s focus on key features by adaptively recalibrating channel features [50]. SA strengthens interactions between features by shuffling and reassembling channel features [51]. These attention mechanisms were integrated to refine the model’s focus and enhance its overall classification performance.

**Fig 2:**
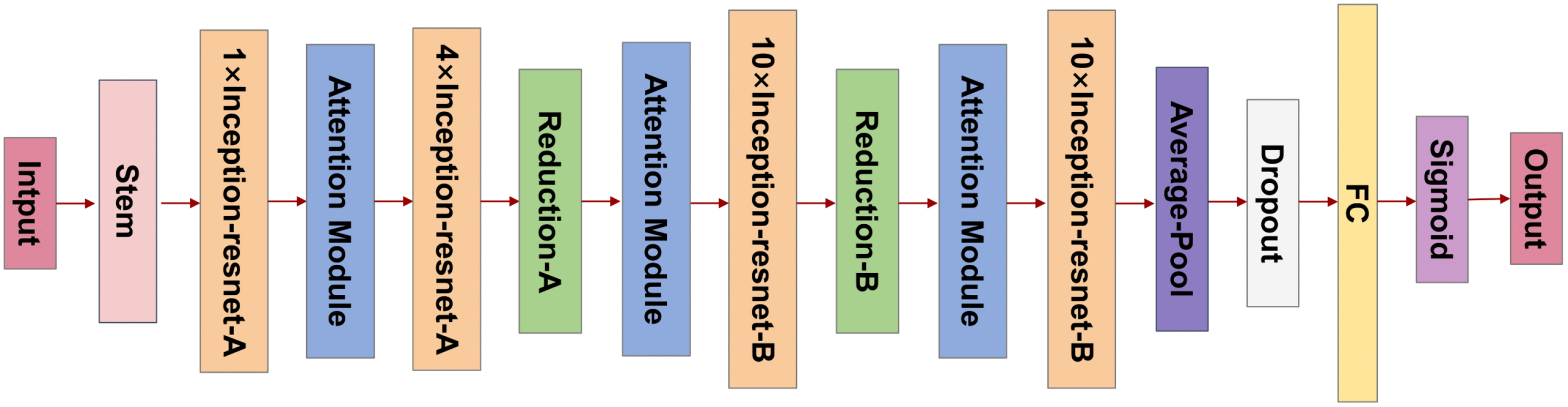
Architecture of inception-ResNetV2 model with integrated attention mechanisms. The architecture of the optimal base model, Inception-ResNetV2, is illustrated. In this model, attention mechanisms were integrated following the first Inception-ResNet-A module, as well as after the Reduction-A and Reduction-B modules. The final model output is generated using a sigmoid activation function, producing the predicted results.

We embedded the above attention mechanism modules at three key positions within the model: after the first Inception-ResNet-A module, after the Reduction-A module, and after the Reduction-B module (the specific model structure is shown in Fig 2). Additionally, to address the issue of class imbalance, we incorporated Focal Loss as the loss function. Focal Loss is an improved cross-entropy loss function that reduces the loss weight for easily classified samples and increases focus on hard-to-classify samples, thereby improving the model’s performance in tasks with class imbalance [52]. Focal Loss introduces a modulating factor ${\left(1-p_{t}\right)}^{\gamma }$and a weighting parameter α to reduce the relative loss contribution from easily classified samples and emphasize hard, minority-class examples. The loss function is defined as:

$L_{focal}=\;-\alpha_{t}{\left(1-p_{t}\right)}^{\gamma }\text{log}\left(p_{t}\right)$,

where 𝑝_𝑡_ denotes the model’s estimated probability for the ground-truth class, 𝛼_𝑡_

∈[0,1]balances class importance, and 𝛾 ≥ 0 controls the focusing strength. In this study, a systematic hyperparameter search was performed with 𝛼 values of 0.25, 0.5, 0.75, and 1.0, and 𝛾 values of 0.5–3.0. Specifically, the weighting factor 𝛼 was varied from 0.25 to 1.0 to cover the full range of class weighting commonly reported in medical imaging tasks, where minority-class proportions typically range between 10–30%. The focusing parameter 𝛾 was tested from 0.5 to 3.0, following the range suggested by Lin et al. [52] and subsequent works, as values within this interval effectively modulate the contribution of easy versus hard samples without causing optimization instability. This range thus ensured both theoretical coverage and empirical relevance for our dataset, allowing a balanced exploration of focusing strength and class weighting effects. The optimal combination (α = 0.75, γ = 2) yielded the best validation AUC and was used in all experiments. This configuration effectively mitigated class imbalance by guiding the model to focus more on difficult and minority-class samples, improving sensitivity for peritoneal metastasis prediction. Comprehensive results for all parameter combinations are presented in S2 Table.

To evaluate the robustness of our final model—the Inception-ResNetV2 model equipped with SE attention and trained on z-score–normalized 40/350 HU images—we conducted a comparative experiment in which images were re-windowed to 30/300, 50/150 and 50/400 HU, and min–max normalized under the default 40/350 HU window. All other steps of the pipeline remained unchanged. These alternative window settings were selected to represent clinically plausible variations around the standard abdominal soft-tissue window (40/350 HU). The 30/300 HU range corresponds to a narrower window commonly used to enhance soft-tissue detail but may saturate high-attenuation regions [53], whereas 50/150 HU and 50/400 HU broaden the window to capture higher-density structures such as vessels or calcifications at the cost of reduced soft-tissue contrast [54]. Evaluating these settings allowed us to systematically examine whether deviations toward narrower or broader intensity ranges would alter the visual distinction between the enhancing gastric wall and surrounding perivisceral fat. Both z-score and min–max normalization were further compared to evaluate the impact of different intensity scaling schemes on network convergence and representation stability.

In addition, the three attention mechanisms that achieved the highest ROC-AUC values in the single-module comparison were permuted across the model’s three attention blocks, yielding six candidate configurations. Each configuration was retrained from scratch and evaluated on the independent test set to determine whether multi-attention combinations could further enhance performance.

We also used the smooth grad algorithm to visualize the model’s focus areas on the CT images. Smooth Grad is an algorithm used to generate more interpretable and visually coherent saliency maps, highlighting the areas of an image that a model focuses on when making predictions. By adding small amounts of random noise to the input data and averaging the resulting gradient maps, Smooth Grad reduces the visual noise and sharpens the salient regions, making it easier to understand and interpret the model’s decision-making process [55]. This approach enhances the reliability of the generated attention maps, providing clearer insights into the features that are most influential in the model’s predictions. During training, the initial learning rate was set at 0.001, with the AdamW optimizer, a batch size of 4, and dropout probabilities set at 0 and 0.3. The deep learning models were implemented using PyTorch 1.12.0, torchvision 0.13.0, and Python 3.9.18. To enhance the reproducibility of the models, all random seeds were set to 42. The computational experiments were conducted using a system equipped with an NVIDIA GeForce RTX 4080 GPU and an Intel Core i7-13700K CPU.

### Performance evaluation

To comprehensively evaluate model performance, we first assessed discrimination with ROC-AUC, complemented by precision–recall curves and the corresponding PR-AUC to account for class imbalance. Pair-wise differences in ROC-AUC (and, where relevant, PR-AUC) between attention mechanisms were formally tested with DeLong’s method. Because the dataset is imbalanced (PM: non-PM ≈ 1: 4.5), ROC-AUC alone may miss clinically meaningful gains; therefore, we additionally reported class-specific operating characteristics—sensitivity, specificity, positive predictive value, and negative predictive value—calculated at the Youden-optimized threshold. Calibration was examined with 500-sample bootstrap calibration curves and quantified by the Brier score and log-loss, while clinical utility was summarized with DCA, expressed as the area under the net-benefit curve (auc-NB) and the maximum net benefit.

All ROC, PR, calibration and DCA plots were generated with Python 3.9.18 (scikit-learn, matplotlib, decision-curve-py). Calibration bootstrapping and DeLong testing were carried out in R 4.3.0 (rms, pROC). Conventional clinical-data statistics were performed in SPSS v22.0. All tests were two-sided, with statistical significance defined as P < 0.05.

## Results

### Clinical characteristics

This study included 585 patients, distributed into training (349), validation (118), and test (118) sets. The mean age was 56.11 years (SD: 11.91), with a composition of 388 males (66.3%) and 197 females (33.7%). The clinicopathological characteristics of the three datasets are listed in Table 1, showed no significant differences in age, gender, tumor location, pathological type, lymph node metastasis, or peritoneal metastasis, confirming comparability and appropriate grouping.

### Performance of various CNN models

**Fig 3:**
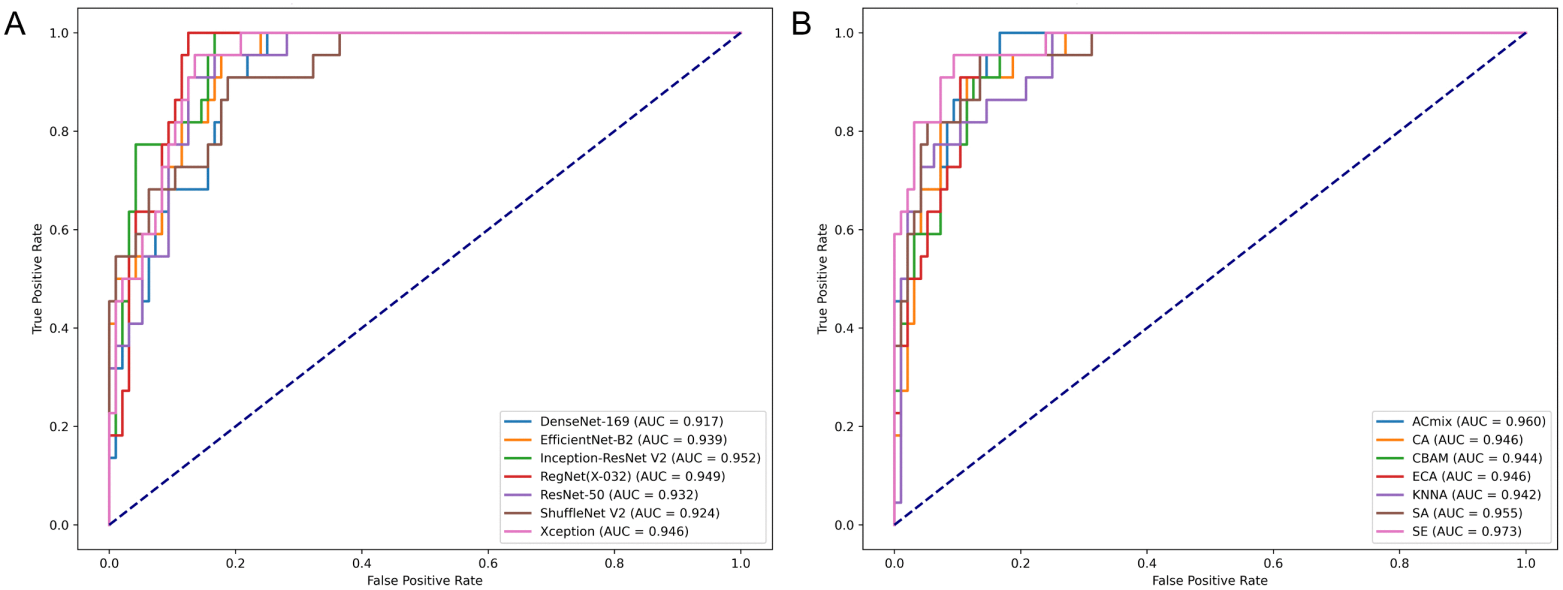
ROC curves of various models on the test set. (A) shows the ROC Curves of various CNN models on the test set; (B) illustrates the ROC Curves of the Inception-ResNetV2 model with different attention mechanisms. ROC, Receiver Operating Characteristic; CNN, Convolutional Neural Network; AUC refers specifically to the area under the ROC curve.

**Fig 4:**
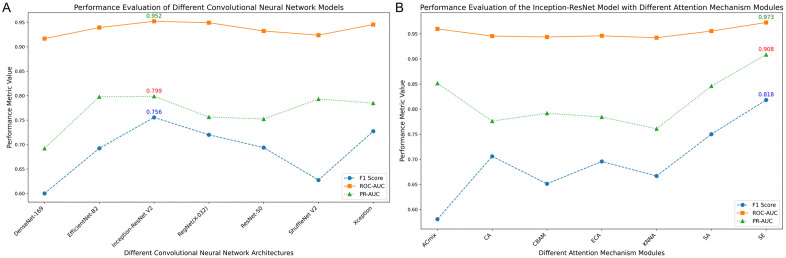
Line plots of performance metrics across various models on test set. **(A)** Figure A shows the performance metrics of various CNN models on the test set; **(B)** Figure B illustrates the performance metrics of the Inception-ResNetV2 model with different attention mechanisms. ROC-AUC, Receiver Operating Characteristic Area Under the Curve; PR-AUC, Precision-Recall Area Under the Curve; CNN, Convolutional Neural Network.

**Fig 5:**
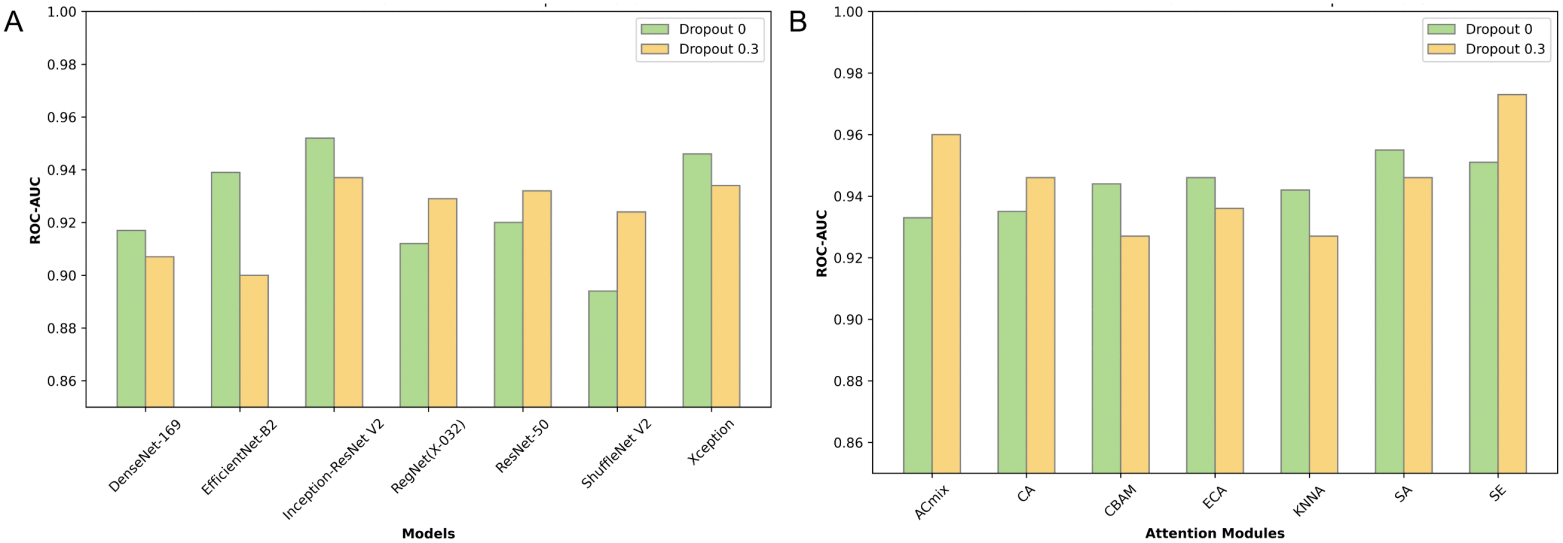
ROC-AUC of various models at different dropout probabilities on test set. **(A)** Figure A shows the ROC-AUC values of different convolutional neural network models at dropout probabilities of 0 and 0.3; **(B)** Figure B displays the ROC-AUC values of the Inception-ResNetV2 model with various attention mechanisms integrated at dropout probabilities of 0 and 0.3. ROC-AUC, Receiver Operating Characteristic Area Under the Curve.

Initially, we evaluated several classical CNN models on ROC-AUC, PR-AUC, and F1-Score, using the test set. The ROC curves are displayed in Fig 3A. The Inception-ResNetV2 model demonstrated superior performance with a ROC-AUC of 0.952. Although DeLong’s test showed no significant differences between the models’ ROC curves, Inception-ResNetV2 also excelled in PR-AUC and F1-Score, achieving 0.799 and 0.756 respectively, as illustrated in Fig 4A. These results led to its selection as the base model for further enhancement. Detailed performance metrics for other models are listed in Table 2, and performance under various dropout probabilities is provided in Fig 5A. Predicted probabilities for test set samples by all base models are shown in S3 Table.

**Table 2:** Performance comparison of various CNN models.

Model	Epoch	Dropout	Precision	Sensitivity	Specificity	F1-Score	ROC-AUC	PR-AUC
DenseNet-169	147	0	0.536	0.682	0.865	0.600	0.917	0.692
EfficientNet-B2	41	0	0.600	0.818	0.875	0.692	0.939	0.798
Inception-ResNetV2	29	0	0.739	0.773	0.938	0.756	0.952	0.799
RegNet(X-032)	123	0.3	0.643	0.818	0.896	0.720	0.949	0.756
ResNet-50	137	0.3	0.630	0.773	0.896	0.694	0.932	0.752
ShuffleNet V2	31	0.3	0.552	0.727	0.865	0.627	0.924	0.793
Xception	53	0	0.606	0.909	0.865	0.727	0.946	0.785

### Enhancement with attention mechanisms

Leveraging the robust Inception-ResNetV2 model as our base, we integrated various attention mechanisms to further enhance performance. The performance enhancements were measured by plotting ROC curves (Fig 3B), and evaluating F1-Score and PR-AUC results (Fig 4B). The SE-enhanced Inception-ResNetV2 model emerged as the top performer, achieving a ROC-AUC of 0.973, a PR-AUC of 0.908, and an F1-Score of 0.818. Detailed results for various models, along with their corresponding dropout probabilities, are presented in Table 3, with additional data on model performance under different dropout settings available in Fig 5B. Predicted probabilities for the test set samples by models incorporating different attention mechanisms are documented in S4 Table.

**Table 3:** Performance comparison of inception-ResNet V2 models with various attention mechanisms.

Modules	epoch	dropout	Precision	Sensitivity	Specificity	F1 Score	ROC-AUC	PR-AUC
ACmix	59	0.3	1.000	0.409	1.000	0.581	0.960	0.851
CA	54	0.3	0.621	0.818	0.885	0.706	0.946	0.776
CBAM	76	0	0.667	0.636	0.927	0.651	0.944	0.792
ECA	36	0	0.667	0.727	0.917	0.696	0.946	0.784
KNNA	58	0	0.857	0.545	0.979	0.667	0.942	0.761
SA	80	0	0.692	0.818	0.917	0.750	0.955	0.846
SE	66	0.3	0.818	0.818	0.958	0.818	0.973	0.908

**Fig 6:**
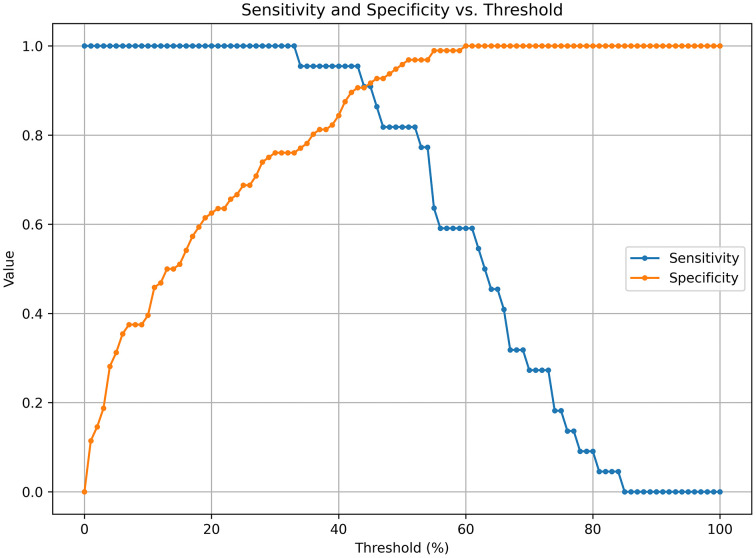
Sensitivity and specificity across different probability thresholds for peritoneal metastasis prediction using the Inception-ResNetV2-SE model. Sensitivity and specificity curves corresponding to various probability thresholds for peritoneal metastasis prediction using the SE-enhanced model. The x-axis represents the probability threshold (%) ranging from 0 to 100, while the y-axis shows the sensitivity (blue line) and specificity (orange line). An optimal threshold of 43% was identified, achieving a sensitivity of 95.5% and a specificity of 90.6%, providing the best balance for clinical decision-making.

**Fig 7:**
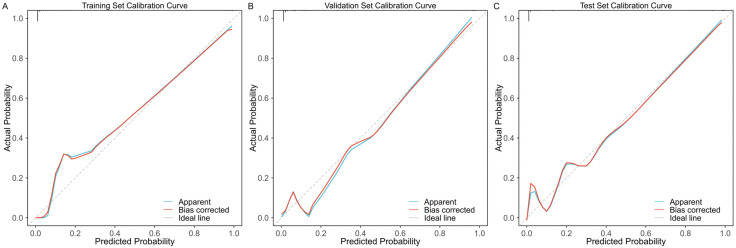
Calibration curves of the SE-enhanced inception-ResNetV2 model across training, validation, and test sets. **(A)** Figure A displays the calibration curve for the test set; **(B)** Figure B displays the calibration curve for the training set; **(C)** Figure C displays the calibration curve for the validation set.

**Fig 8:**
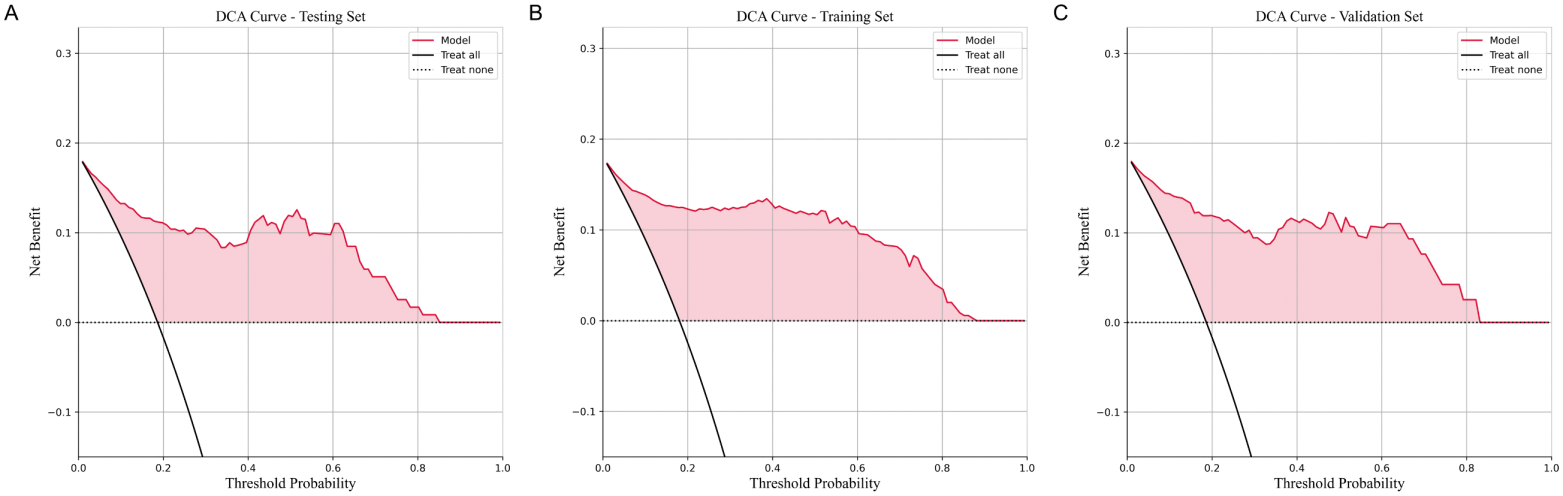
Decision curve analysis curves of the SE-enhanced inception-ResNetV2 model across training, validation, and test sets. **(A)** Figure A displays the DCA curve for the test set; **(B)** Figure B displays the DCA curve for the training set; **(C)** Figure C displays the DCA curve for the validation set. DCA, Decision Curve Analysis.

**Fig 9:**
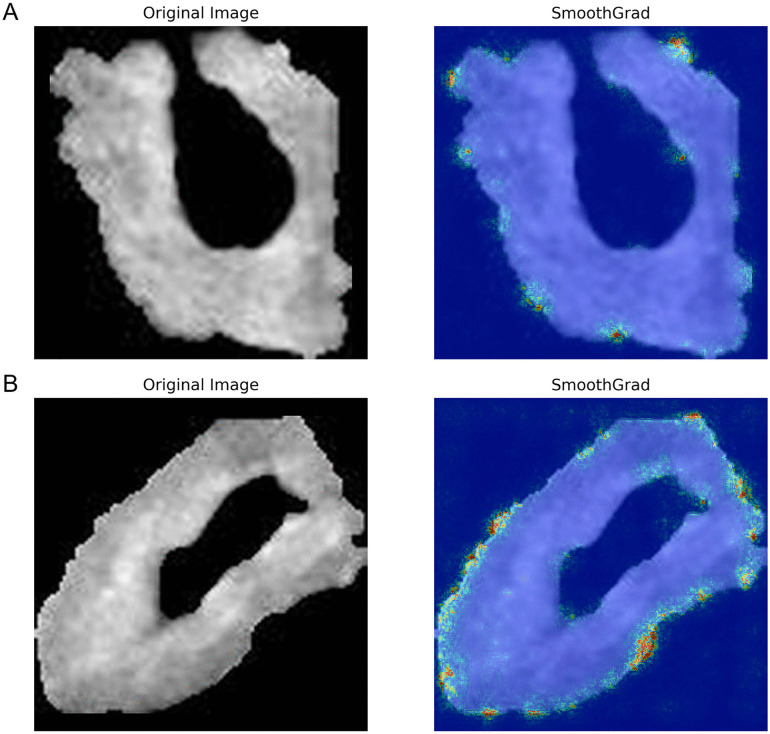
Smooth grad visualization of model attention on tumor regions. Figure A provides the visualization for a gastric cancer patient without peritoneal metastasis; Figure B showcases the visualization for a patient with peritoneal metastasis.

The SE-enhanced model achieved an initial sensitivity of 81.8% and a specificity of 95.8%. Through a systematic evaluation of sensitivity and specificity across probability thresholds from 0% to 100%, the optimal threshold was determined to be 43%, yielding a maximum combined sensitivity (95.5%) and specificity (90.6%). As shown in Fig 6, selecting this threshold optimally balances sensitivity and specificity, thereby improving the model’s overall diagnostic performance. The sensitivity and specificity values for each probability threshold are provided in S5 Table. Furthermore, the calibration curve indicated that the SE-optimized model demonstrated good calibration between predicted and observed outcomes (Fig 7). DCA further revealed that the model provided substantial clinical benefit across various risk thresholds (Fig 8). Visualization analysis using the Smooth Grad algorithm showed that the model’s attention in the CT images’ ROI was primarily focused on the tumor margins, suggesting that imaging features near the tumor edges play a crucial role in the model’s discriminative ability (Fig 9). Beyond diagnostic performance, we evaluated the model’s computational efficiency. The Inception-ResNetV2-SE model consists of approximately 55 million parameters, leading to a relatively longer training time per epoch compared to lighter architectures. Nevertheless, the inference speed remained clinically viable, with a validation time of approximately 1 second for four CT images, supporting its feasibility for near real-time application. A comprehensive comparison of computational efficiency across different models is presented in S6 Table.

### Comprehensive comparison of attention mechanisms

Pair-wise DeLong’s tests showed no statistically significant ROC-AUC differences among attention modules (P > 0.05). Nevertheless, the SE module achieved the lowest Brier score (0.079) and log-loss (0.278), the highest decision-curve net benefit (auc-NB = 0.079) (S7 Table). These findings indicate superior calibration and clinical utility of the SE architecture despite comparable ROC-AUC values.

Permuting the three top‐ranked attention modules across the network’s three blocks produced six ensemble configurations; none outperformed the single SE-SE-SE model on any metric, with ROC-AUC differences ＞0.03 and all DeLong P values < 0.05, indicating that channel-wise recalibration alone offers the most synergistic effect throughout the model (S8 Table).

When the final Inception-ResNetV2-SE network was re-evaluated on the four alternative pre-processing variants from this comparative experiment, ROC-AUC dropped to 0.878–0.939 and all DeLong P values versus the baseline were < 0.05, confirming that the clinically recommended 40/350 HU window combined with z-score normalization remains optimal (S9 Table).

## Discussion

In this study, we conducted a systematic exploration of building predictive models for peritoneal metastasis in gastric cancer patients by comparing various CNN models and incorporating attention mechanism modules. The Inception-ResNetV2 model demonstrated consistently strong performance, making it the optimal choice for further optimization. In subsequent research, we enhanced the Inception-ResNetV2 model by adding various attention mechanism modules to further improve its performance. The results demonstrated that the Inception-ResNetV2 model with the SE module achieved favorable results across multiple complementary metrics. Therefore, we ultimately identified the Inception-ResNetV2-SE model as the final model for predicting the presence of peritoneal metastasis in T3/T4 stage patients. This model exhibited a sensitivity of 81.8%, a specificity of 95.8%, a ROC-AUC value of 0.973, a PR-AUC value of 0.908, and an F1-score of 0.818.

Pair-wise DeLong tests revealed no statistically significant ROC-AUC differences among the three attention variants (all P > 0.05). This lack of significance is expected given the small, imbalanced test set (PM: non-PM ≈ 1: 4.5) and the near-ceiling ROC-AUC values of all models (> 0.94), which leave inter-model gaps (< 0.02) too narrow to achieve P < 0.05 without substantially larger positive samples [56,57]. Nevertheless, the Inception-ResNetV2-SE model consistently outperformed its competitors on metrics that better reflect clinical relevance under class imbalance: it achieved the most favorable calibration (Brier = 0.079; log-loss = 0.278), the highest net benefit across clinically pertinent thresholds (auc-NB = 0.079), and the greatest precision (0.82) at recall ≥ 0.80. These advantages justify selecting the SE variant as the final predictive model and align with current recommendations to complement ROC-based comparisons with calibration and decision-analytic metrics when validating AI tools for high-stakes clinical use [58–60].

In clinical practice, the probability threshold can be set at 0.43, indicating that peritoneal metastasis is predicted when the model’s output probability surpasses this value. At this threshold, the model attains a sensitivity of 95.5% and a specificity of 90.6%, achieving an optimal trade-off between accurately identifying true positive cases and minimizing false positives. Given this high diagnostic accuracy, integrating the model into clinical workflows could substantially reduce unnecessary staging laparoscopies, thereby significantly lowering associated healthcare costs and patient burden. Specifically, patients with a model-predicted probability above 43% would undergo staging laparoscopy for confirmation, while those below the threshold could avoid this invasive procedure. Such targeted utilization of staging laparoscopy is essential for detecting clinically occult peritoneal metastases not visible through conventional imaging, preventing unnecessary non-curative surgeries, and thus optimizing individualized treatment strategies for patients with T3/T4-stage gastric cancer [61,62]. Ultimately, implementing this predictive model could facilitate earlier and more precise identification of high-risk patients, enable timely therapeutic interventions, and enhance overall clinical and economic efficiency.

Our study differentiates itself from previous research by systematically incorporating attention mechanisms into CNN architectures specifically optimized for T3/T4-stage gastric cancer. Unlike Zhou et al.[63], who employed machine learning models based solely on clinical parameters, our approach incorporates imaging-based deep learning, enabling more comprehensive feature extraction and significantly improving predictive performance (ROC-AUC: 0.973 vs. 0.745). Moreover, in contrast to Mirniaharikandehei et al. [18], who depended on handcrafted radiomic features and conventional machine learning classifiers, our end-to-end deep learning framework obviates manual feature selection and achieves a substantial gain in sensitivity (81.8% vs. 43.1%) while preserving high specificity. This underscores the advantage of automated deep feature learning in capturing subtle imaging biomarkers associated with peritoneal metastasis. Additionally, Jiang et al. applied a densely connected CNN without explicit attention mechanisms, achieving a ROC-AUC of 0.933 [17]. More recently, Zhu et al. developed a machine learning-based CT radiomics nomogram for OPM prediction in advanced gastric cancer, with the integrated radiomics model achieving an AUC of 0.835 in the test set [64]. Zou et al. further proposed a multicenter cascaded segmentation-classification DL framework integrating V-Net-based tumor segmentation with metastatic risk classification, achieving an AUC of 0.916 in the internal OPM test cohort [65]. By integrating Squeeze-and-Excitation (SE) attention modules into a unified CNN framework, our model enhances channel-wise feature recalibration and improves the representation of subtle CT imaging patterns associated with peritoneal metastasis. Compared with previous clinical-parameter-based machine learning models, handcrafted radiomics-based classifiers, and cascaded segmentation-classification deep learning frameworks, our approach provides an end-to-end attention-enhanced model specifically optimized for high-risk T3/T4-stage gastric cancer patients. In this setting, our model achieved a ROC-AUC of 0.973, exceeding the reported performance of the above approaches. This design reduces dependence on clinical variables or handcrafted feature engineering and enables systematic evaluation of attention mechanisms within the same CNN backbone. Therefore, our study offers a more task-specific predictive framework for preoperative PM assessment, potentially improving risk stratification and reducing unnecessary surgical interventions.

In this study, we conducted a visualization analysis of the final model’s attention areas using the Smooth Grad algorithm. The results showed that the model primarily focused on the tumor margins within the CT images’ ROI. Consistent with prior studies, this observation suggests that subtle peripheral variations in gastric tumors offer valuable predictive information for assessing peritoneal metastasis, thus providing additional insight into the model’s decision-making mechanism [61,66].

During the model selection phase, after comparing the performance of various CNN models, we ultimately selected the Inception-ResNetV2 model. This model uniquely combines the Inception module’s ability to extract features at multiple scales with ResNet’s efficient residual blocks, effectively mitigating the problem of vanishing gradients in deep networks. The Inception module’s ability to extract features across multiple scales allows the model to capture complex and richly layered information within images, which is particularly crucial when processing complex medical imaging data. Additionally, ResNet’s residual structure introduces skip connections, enabling deeper network training while significantly enhancing stability and convergence speed [38].

Building on this foundation, we further explored the possibility of enhancing model performance by adding different attention mechanism modules. These modules were embedded at three different positions within the Inception-ResNetV2 model: after the first Inception-ResNet-A module, after the Reduction-A module, and after the Reduction-B module. This approach not only enhanced feature representation across different network layers but also optimized the entire feature extraction process, allowing the model to benefit from attention mechanisms at every stage. In our experiments, we evaluated several attention modules, including ACmix, CBAM, CA, ECA, KNNA, SE, and SA, to determine their effectiveness in enhancing feature representation for predicting peritoneal metastasis in T3/T4 stage gastric cancer patients. Our comparative evaluation revealed that incorporating the SE module substantially improved overall model performance. Although improvements in ROC-AUC did not reach statistical significance, the Inception-ResNetV2 model integrated with the SE module demonstrated comprehensive enhancements across other evaluation metrics, particularly in scenarios involving imbalanced datasets. The adaptive weighting mechanism employed by the SE module effectively enhanced the model’s capability to selectively emphasize critical feature maps, thereby increasing the precision and reliability of peritoneal metastasis predictions [50]. Our findings underscore the importance of channel-wise recalibration in boosting the sensitivity and specificity of deep learning models for medical imaging. In contrast, modules such as CBAM and SA, which simultaneously incorporate spatial and channel attention, aim to capture extensive spatial-channel relationships. ACmix and KNNA utilize more complex transformations that, despite their potential for enriched feature representation, are prone to overfitting and parameter convergence issues, particularly on smaller, imbalanced medical imaging datasets. Modules like CA and ECA, while offering efficient mechanisms to emphasize critical features, still introduce structural complexity that can hinder performance compared to the simpler, channel-focused SE module. The SE module’s streamlined and effective channel recalibration strategy directly adjusts individual feature map weights, striking an optimal balance between performance and complexity, thus mitigating risks of overfitting and ensuring stable model convergence.

When we explored inserting different attention mechanisms at the three predefined sites of Inception-ResNetV2, all six heterogeneous combinations of the top-ranked modules (SE, SA, ACmix) yielded lower ROC-AUCs than the uniform SE–SE–SE architecture (all DeLong P < 0.05). Several factors may explain this observation. First, SE delivers pure channel-wise recalibration, which appears optimally aligned with the predominantly intensity-driven cues of venous-phase gastric CT [67]; mixing in spatial or hybrid modules (SA, ACmix) may introduce redundant or noisy spatial gating that dilutes these signals [68]. Second, employing the same attention module at each depth maintains a uniform recalibration strategy that keeps feature distributions and gradient flow consistent, whereas mixing different modules both disrupts this continuity and introduces additional parameters—complications that a small, imbalanced dataset (~585 cases; PM: non-PM ≈ 1: 4.5) cannot reliably optimize, thereby heightening the risk of over-fitting [69,70]. Collectively, these factors suggest that applying the same lightweight channel attention at each critical stage offers the most synergistic and data-efficient enhancement for this specific task.

A comparative experiment on window settings and intensity normalization showed that image contrast and scaling materially affect model performance. Re-windowing the CT scans away from the clinically recommended 40/350 HU to 30/300, 50/150 or 50/400 HU reduced ROC-AUC to 0.878–0.939, and all DeLong P values versus the baseline were < 0.05. Similarly, substituting min–max normalization for z-score under the default window lowered ROC-AUC to 0.923 and produced parallel declines in PR-AUC and F1-score. Unlike z-score— which centers voxel intensities around the global mean and scales by the standard deviation, thereby preserving relative contrast above and below the mean—min–max squeezes the entire range into 0 ~ 1, attenuating subtle intensity differences and saturating outliers. This compression flattens the gradients available to the network and weakens the channel-recalibration signal exploited by the SE module [71]. The results are physiologically plausible: the 40/350 HU window maximizes contrast between enhancing gastric wall and perivisceral fat, whereas narrower windows saturate soft-tissue intensities and broader windows dilute local contrast [72]. Combined with min–max scaling, such windows further homogenize voxel distributions and erode the discriminative information that attention mechanisms rely on. These findings underscore that appropriate windowing and statistical normalization are not cosmetic choices but key determinants of downstream deep-learning performance, especially for attention-based architectures that depend on fine-grained intensity gradients.

From a practical perspective, computational efficiency is a key consideration for clinical translation of AI models. Although the Inception-ResNetV2-SE network contains approximately 55 million parameters, its inference time of roughly one second for four CT slices remains compatible with real-time or near–real-time decision support in a clinical workflow. This efficiency–accuracy balance suggests that high-capacity architectures can be feasibly deployed on modern GPUs without prohibitive latency. Nevertheless, future work may explore model compression, pruning, or knowledge distillation strategies to further reduce computational overhead and facilitate integration into routine radiology systems.

Despite the encouraging results of this study, there are some limitations. First, this is a single-center retrospective study. Although we divided the data into independent training, validation, and test sets, we did not use data from other hospitals or regions, so the generalizability of the model remains to be validated. To improve transparency and facilitate reproducibility, a de-identified subset of cropped venous-phase CT tumor images and the final model implementation code have been made publicly available on GitHub, enabling other researchers to reproduce and extend our work. Future studies should include external multicenter validation to further confirm the robustness and clinical applicability of the model. Second, the relatively small dataset, although reasonable for medical AI studies, may constrain the statistical power for detecting subtle performance differences among methods. Moreover, there was a noticeable class imbalance, with the ratio of peritoneal metastasis to non-metastasis cases being approximately 1:4.5. Although we applied multiple strategies—such as extensive data augmentation, the use of Focal Loss to emphasize minority-class samples, and stratified sampling during cross-validation—to mitigate this imbalance, it may still have affected the model’s overall performance and stability. As larger and more diverse datasets become available through ongoing multi-center collaborations, advanced data synthesis or oversampling techniques (e.g., Generative Adversarial Networks) could be further explored to alleviate class imbalance and enhance the robustness of model training [73]. Third, the ROIs in this study were manually delineated by radiologists. For clinical practice, high-performance automatic delineation algorithms would need to be developed to improve the model’s practicality. However, due to the significant variability in gastric tumors and their low contrast with normal tissue, achieving high-precision automatic segmentation remains challenging. Future work could explore the integration of deep-learning-based segmentation models to automate ROI selection, thereby reducing inter-observer variability and enhancing reproducibility. Fourth, this study primarily relied on 2D CT images, even though 3D ROIs can offer a more comprehensive representation of tumor characteristics. Currently, adopting 3D ROIs in deep learning models faces several challenges, including effective handling of complex structures, substantially higher computational resource demands, and large data requirements [74,75]. Fifth, as with most deep learning–based models, our approach inherently functions as a “black-box” system. Although we employed Smooth Grad visualization to partially elucidate the model’s attention to peritumoral regions, the internal decision-making process remains only partially interpretable. This limitation may reduce clinical transparency and hinder physicians’ confidence in AI-assisted decision-making. Future work should integrate explainable AI techniques—such as feature attribution maps, or attention interpretability frameworks—to provide more transparent reasoning and facilitate clinical acceptance. Moreover, for gastric cancer, robust 3D automatic segmentation algorithms remain under development [76], which hinders the direct integration of 3D approaches into routine clinical workflows. A promising avenue for future research lies in designing hybrid 2D–3D architectures that leverage volumetric data while preserving computational efficiency [77]. Additionally, developing and integrating more advanced, reliable tumor automatic segmentation algorithms will be crucial for translating these 3D-based methods into practical clinical applications. Despite the strong performance of our model, some limitations persist. Following probability threshold adjustment, the model exhibited false-negative and false-positive rates of 4.5% and 9.4%, respectively. Thus, its predictions should be integrated with other diagnostic modalities, such as endoscopic ultrasound and biomarkers, to enhance decision-making for staging laparoscopy. Future research should explore comprehensive multimodal integration by incorporating radiomic features, clinical parameters, blood-based biomarkers, genomic alterations, and histopathological findings. This holistic approach has the potential to further enhance the model’s predictive performance, increase its robustness, and improve its overall clinical applicability. Future prospective studies will be crucial for evaluating the clinical applicability of our model and validating its real-world impact on clinical decision-making, thereby ensuring its effectiveness in routine practice.

## Conclusion

In summary, this study systematically assessed the efficacy of various deep learning models for predicting clinically occult peritoneal metastasis in T3/T4 stage gastric cancer patients based on CT images. The results demonstrated that the Inception-ResNetV2-SE model outperformed others across multiple evaluation metrics. The reliability of this model suggests that using it to identify high-risk patients could significantly reduce unnecessary surgical interventions through targeted staging laparoscopy, thereby enhancing patient outcomes. This study not only extends the application of deep learning techniques in medical image analysis but also provides a new pathway for precision diagnosis and treatment of gastric cancer patients.

## Supporting information

S1 Table Computed tomography protocol of the three machines. This table includes details such as slice thickness, contrast agent dosage, acquisition time, and other relevant imaging parameters. These protocols were standardized across machines to ensure consistency in image quality for preoperative assessment of T3/T4 stage gastric cancer patients.
(XLSX)

S2 Table Performance of different Focal Loss parameter combinations for peritoneal metastasis prediction using the Inception-ResNetV2-SE model. This table presents the training and validation performance of the Inception-ResNetV2-SE model under different Focal Loss parameter settings. The α (alpha) and γ (gamma) values were varied to identify the optimal combination for model performance. The best epoch represents the training epoch with the highest validation ROC-AUC. The Train ROC-AUC and Val ROC-AUC columns indicate the area under the receiver operating characteristic curve for the training and validation datasets, respectively. Based on these results, α = 0.75 and γ = 2 achieved the highest validation ROC-AUC (0.9531), making it the optimal choice for model training.
(XLSX)

S3 Table Prediction probabilities of different convolutional neural network models at different dropout probability on the test set. This table displays the prediction probabilities of different classical CNN models for peritoneal metastasis in test set samples at dropout probabilities of 0 and 0.3.
(XLSX)

S4 Table Prediction probabilities of Inception-ResNetV2 model with different attention mechanisms at different dropout probability on the test set. This table displays the prediction probabilities of the Inception-ResNetV2 model with different attention mechanisms for peritoneal metastasis in test set samples at dropout probabilities of 0 and 0.3.
(XLSX)

S5 Table Sensitivity and specificity of the Inception-ResNetV2-SE model at different probability thresholds on the test set. This table presents the sensitivity and specificity of the Inception-ResNetV2-SE model across different probability thresholds for peritoneal metastasis prediction on the test set. The Threshold (%) column represents the probability cutoff used for classification, while the Sensitivity and Specificity columns indicate the model’s corresponding performance metrics. The total column reflects the combined sensitivity and specificity score for each threshold. At a probability threshold of 0.43, the model achieved the optimal balance, with a sensitivity of 95.5% and a specificity of 90.6%, making it the most suitable threshold for clinical decision-making.
(XLSX)

S6 Table Total parameters, training time, and inference time of different models and attention-enhanced Inception-ResNetV2 variants. This table summarizes the total parameter count, epoch training time, and epoch validation (inference) time for various deep learning models and Inception-ResNetV2 integrated with different attention mechanisms. The Inception-ResNetV2 model enhanced with the SE module exhibited a relatively higher parameter count (55.0M) and longer training time (17s per epoch) compared to other architectures. However, its inference time remained clinically feasible (~1s per epoch), making it suitable for real-time application in clinical settings. These findings highlight the trade-off between model complexity and computational efficiency, emphasizing the importance of balancing performance and practicality for clinical deployment.
(XLSX)

S7 Table DeLong P values, calibration metrics, and net-benefit indices for each attention module versus the SE baseline. This table contrasts the final SE model with six alternative attention modules (ACmix, CA, SA, KNNA, ECA, CBAM). Pair-wise DeLong tests showed no statistically significant ROC-AUC differences between the SE module and any alternative attention module (P > 0.05). Nevertheless, the Inception-ResNetV2 model equipped with the SE module achieved the lowest Brier score and log-loss and the highest decision-curve metrics (auc-net-benefit and maximum net benefit), indicating superior calibration and clinical utility relative to the other attention mechanisms.
(XLSX)

S8 Table Test-set performance of Inception-ResNetV2 models with heterogeneous attention-module permutations compared with the uniform SE–SE–SE baseline. This table reports ROC-AUC, PR-AUC, F1-score, sensitivity, specificity and accuracy for six candidate configurations obtained by permuting the three top-ranked attention mechanisms (SE, SA and ACmix) across the network’s three attention blocks. The rightmost column lists DeLong P values for pair-wise ROC-AUC comparisons with the SE–SE–SE model. All heterogeneous permutations showed significantly lower ROC-AUC (P < 0.05), indicating that the channel-wise recalibration provided by the SE module alone yields the most effective attention strategy.
(XLSX)

S9 Table Test-set performance of the Inception-ResNetV2-SE model under alternative CT window settings and normalization methods compared with the 40/350 HU z-score baseline. The table lists discrimination (AUROC, PR-AUC), threshold-based (F1-score, sensitivity, specificity, accuracy) and DeLong P values for four preprocessing variants—three alternative window widths/levels (30/300, 50/150, 50/400 HU) and min-max normalization under the default window. All variants yielded significantly lower AUROC than the baseline (P < 0.05), confirming that the standard 40/350 HU window combined with z-score normalization provides the most favorable contrast and predictive performance.
(XLSX)
